# Comparative proteomic responses of *Paranosema locustae* spores to desiccation and ultraviolet irradiation

**DOI:** 10.1128/spectrum.01803-26

**Published:** 2026-09-30

**Authors:** Na Zhang, Weiqi Guo, Tinghao Zhang, Hanye Kang, Hangyue Zhang, Liang Yuan, Shuchen Guo, Ye Xu, Rong Ji, Hongxia Hu

**Affiliations:** 1 College of Life Sciences, Xinjiang Key Laboratory of Special Species Conservation and Regulatory Biology, International Research Center of Cross-Border Pest Management in Central Asia, Xinjiang Normal University 12615, Urumqi, Xinjiang Province, China; 2 Tacheng, Research Field (Migratory Biology), Observation and Research Station of Xinjiang, Xinjiang, China; 3 HeNan Province Plant Protection and Quarantine Station, Henan, China; Barnard College, New York, New York, USA

**Keywords:** *Paranosema locustae*, desiccation stress, ultraviolet irradiation, quantitative proteomics, dormancy, stress adaptation

## Abstract

Environmental persistence is essential for the efficacy of the microsporidian parasite *Paranosema locustae* (*P. locustae*), yet the molecular mechanisms underlying its tolerance to desiccation and ultraviolet (UV) stress remain poorly understood. Here, we employed data-independent acquisition (DIA)-based quantitative proteomics to characterize the global proteomic responses of *P. locustae* spores subjected to desiccation and UV irradiation. Spores were exposed either to desiccation at 40°C and ≤30% relative humidity for 5 days or to UV irradiation (≥100 μW/cm²) for 24 h, followed by comparative proteomic analyses against untreated controls. In total, 187 proteins were identified across all treatments. Desiccation stress resulted in 22 upregulated and 8 downregulated proteins, whereas UV irradiation induced 31 upregulated and 12 downregulated proteins. Functional enrichment analyses revealed that differentially expressed proteins were predominantly associated with vacuolar/endosomal trafficking (KEGG sce04145/spo04145), transmembrane transport, and protein homeostasis, while proteins involved in biosynthetic metabolism were generally downregulated. Under desiccation, the proteomic response was characterized by upregulation of vacuolar-trafficking and transmembrane transport proteins together with downregulation of biosynthetic pathways, consistent with entry into a metabolically repressed, dormancy-like state. In contrast, UV irradiation triggered a coordinated stress-response program characterized by enhanced protein processing in the endoplasmic reticulum, proteasomal activity, and the integrated stress response, accompanied by repression of energetically costly processes such as translation and biosynthesis. Together, these findings demonstrate that *P. locustae* deploys distinct, stress-specific molecular responses to desiccation and UV stress.

**IMPORTANCE:**

Environmental persistence is essential for *Paranosema locustae* as a biocontrol agent, with desiccation adaptation helping to elucidate its underlying mechanism; our results demonstrate that desiccation helps maintain *P. locustae* in a dormant state characterized by transcriptional remodeling but suppressed metabolism, while ultraviolet (UV) exposure activates proteostasis and antioxidant pathways and downregulates energy-intensive biosynthesis, thereby providing insights for optimizing microsporidian biocontrol strategies in pest management.

## INTRODUCTION

Microsporidia are obligate intracellular fungal parasites capable of infecting a diverse range of invertebrates and vertebrates (1, 2). To survive outside the host, these parasites rely on highly specialized, dormant spores characterized by a robust three-layered cell wall (3). The proteinaceous exospore, in particular, provides critical physical resistance, allowing spores to persist under environmental extremes (4). Among these parasites, *Paranosema locustae* (*P. locustae*) is a premier microbial agent for locust biocontrol and remains the only commercialized microsporidian product globally (5). Its capacity for both horizontal and vertical transmission enables sustained suppression of locust populations while maintaining high environmental safety.

However, the efficacy of *P. locustae* in field applications is severely constrained by environmental stressors. During large-scale deployment, particularly in summer locust outbreak season, spores are frequently exposed to intense desiccation and ultraviolet (UV) radiation (6). While previous studies have documented the impact of such stressors on pathogenicity, the underlying molecular mechanisms—specifically how these “genomically compact” parasites sense and resist abiotic stress under nutrient-limited conditions—remain poorly understood (6–8). Whether *P. locustae* employs dynamic proteomic regulation to maintain homeostasis under desiccation and UV stress is a critical knowledge gap that limits the optimization of more stable biocontrol formulations.

Building on our previous finding that *P. locustae* exhibits distinct responses to thermal desiccation and UV exposure (6, 9), this study employed data-independent acquisition (DIA)-based quantitative proteomics to systematically analyze the spore proteome. By comparing differentially expressed proteins (DEPs) under desiccation (40°C, relative humidity [RH] ≤ 30%) and high-intensity UV irradiation (≥100 μW/cm²), we aimed to elucidate the proteomic responses of *P. locustae*. Specifically, we investigated the changes in abundance of stress-responsive proteins and the activation of DNA repair machineries. These findings provide novel insights into the evolutionary resilience of microsporidia and offer a theoretical foundation for enhancing the stability and persistence of microbial pesticides.

## MATERIALS AND METHODS

### Spore preparation and rough purification

*P. locustae* spores were provided by the College of Plant Protection, China Agricultural University, and transported to the laboratory on ice. To ensure sample purity, the spore suspension was filtered through six layers of medical gauze to remove locust tissue debris. Following purification, spore concentration was determined using a hemocytometer and adjusted to a final concentration of 1 × 10^9^ spores/mL. The suspension was then aliquoted into 1.5 mL microcentrifuge tubes and stored at −20°C for subsequent experiments.

### Stress treatments

Prior to stress exposure, 1 mL aliquots of the spore suspension (1 × 10^9^ spores/mL) were washed three times with deionized water, following established protocols (6, 9).

Desiccation stress (40°C): spore suspensions were aseptically applied to sterile filter paper and allowed to air-dry at room temperature. Once dried, the spores were transferred to 1.5 mL tubes containing silica gel desiccant to maintain an RH ≤30%. These samples were then incubated at 40°C for 5 days.

Ultraviolet (UV) irradiation: aliquots of the spore suspension were placed in uncovered 6 cm Petri dishes and exposed to UV radiation (253.7 nm) using two 15 W lamps (Jiangsu Juguang Photoelectric Technology Co., Ltd., Suzhou, China) positioned 30 cm above the samples. Irradiation was conducted continuously for 24 h at room temperature (RH ≤ 30%) with a constant intensity of ≥100 μW/cm².

Three independent biological replicates were performed for each treatment group (control, desiccation, UV). Each replicate consisted of spores from independently prepared suspensions.

It should be noted that the UV-C irradiation (253.7 nm) used in this study differs substantially from natural solar UV radiation, which primarily comprises UV-A (315–400 nm) and UV-B (280–315 nm) wavelengths. UV-C is almost entirely absorbed by the Earth’s ozone layer and does not reach the ground under normal atmospheric conditions. However, UV-C was selected here as a standardized laboratory stressor to elicit reproducible proteomic responses and to simulate extreme environmental scenarios, such as high-altitude UV exposure or artificial UV disinfection treatments. While the specific molecular targets may differ between UV-C and solar UV, the conserved stress-response pathways activated—including proteostasis maintenance, antioxidant defense, and translational repression—are likely to be relevant across UV wavelengths. Future field studies should validate whether similar proteomic signatures are observed under natural solar UV exposure to confirm the ecological relevance of these findings for optimizing *P. locustae*-based biocontrol applications.

### Protein extraction and enzymatic digestion

Total protein was extracted using SDT lysis buffer (2.5% SDS, 100 mM Tris-HCl [pH 7.6], 100 mM dithiothreitol [DTT], 1 mM PMSF, ddH_2_O). Samples were homogenized and subjected to ultrasonic disruption, followed by boiling for 15 min to ensure complete denaturation. The crude lysate was clarified by centrifugation at 14,000 × *g* for 15 min, and the protein concentration in the supernatant was determined using a BCA Protein Assay Kit (Beyotime Biotech Inc., Shanghai, China). Protein digestion was performed according to the Filter-Aided Sample Preparation protocol. Briefly, 200 μg of protein from each sample was reduced with DTT and alkylated with iodoacetamide in the dark. The protein mixture was transferred to a 10k or 30k ultrafiltration tube and washed three times with urea-containing buffer to remove detergents. Digestion was carried out overnight at 37°C using sequencing-grade trypsin (Promega, Madison, WI, USA) at a protease-to-protein ratio of 1:50 (wt/wt). The resulting peptides were collected by centrifugation, desalted using C18 cartridges (Empore, Merck KGaA, Darmstadt, Germany), and concentrated by vacuum centrifugation. Finally, the lyophilized peptides were reconstituted in 0.1% (vol/vol) formic acid, and the peptide concentration was quantified by OD_280_ for subsequent liquid chromatography-tandem mass spectrometry (LC-MS/MS) analysis.

### LC-MS/MS analysis and DIA proteomics

LC-MS/MS was performed on an Orbitrap Astral mass spectrometer coupled with a Vanquish Neo UHPLC system (Thermo Fisher Scientific, Waltham, MA, USA). Peptides from each sample were loaded onto a column (μPAC Neo High Throughput column, Thermo Scientific) at a flow rate of 2.2 μL/min for sample loading and column equilibration. The mobile phase A was 0.1% formic acid in water, and B was 0.1% formic acid in 80% acetonitrile. Peptides were eluted over a total gradient time of 8 min at a flow rate of 1.25 μL/min, which was reduced from the loading flow rate to improve electrospray ionization efficiency and chromatographic resolution during gradient separation. The linear gradient was set as follows: 0–0.1 min, B from 4% to 6%; 0.1–1.1 min, B from 6% to 12%; 1.1–4.3 min, B from 12% to 22.5%; 4.3–6.1 min, B from 22.5% to 45%; 6.1–8 min, B maintained at 99%. The eluted peptides were analyzed on an Orbitrap Astral mass spectrometer. The DIA method consisted of a survey scan from 380–980 *m*/*z* at a resolution of 240,000 with an AGC target of 500% and 5 ms maximum injection time. The DIA MS/MS scans were acquired by Astral from 150 to 2,000 *m*/*z* with a 2 *m*/*z* isolation window and with an AGC target of 500% and 3 ms maximum injection time. Normalized collision energy was set to 25%, and cycle time was 0.6 s. The Orbitrap Astral mass spectrometer features a dual-ion-path configuration that enables parallel ion injection and analysis, effectively decoupling the maximum injection time per window from the total cycle time and allowing a 0.6 s cycle across approximately 300 windows. The spectra of full MS scan and DIA scan were recorded in profile and centroid type, respectively.

### Data processing and protein quantification

The DIA MS data were analyzed using DIA-NN 1.8.1. MS data were searched against a custom protein database of 3,863 entries composed predominantly of *Antonospora locustae* protein sequences from NCBI (3,828 entries; NCBI-Antonospora locustae [278021]-3863-20241014.fasta), supplemented with 30 Swiss-Prot sequences from the closely related microsporidian Enterocytozoon bieneusi, two PDB structural entries, and three additional RefSeq entries. The database search parameters were enzyme, trypsin; maximum missed cleavages, 1; fixed modification, carbamidomethylation (C); variable modifications, oxidation (M) and acetylation (protein N-term); precursor mass tolerance, 10 ppm; fragment mass tolerance, 10 ppm; peptide length range, 7–30; peptide charge range, 1–4; fragment *m*/*z* range, 150–2,000; FDR threshold, <1% at both PSM and protein level. Differentially expressed proteins were defined as those with a fold change (FC) ≥1.5 or ≤0.67 and a *P* value < 0.05, as determined by a two-tailed Student’s *t*-test comparing each treatment group to the control.

### Bioinformatics and statistical analysis

Bioinformatic analyses were performed using the R statistical computing environment (version 4.2.2). DEPs were visualized through hierarchical clustering and volcano plots generated by R packages (clusterProfiler v4.0). Functional annotation of the identified proteins was conducted against the UniProtKB/Swiss-Prot, Kyoto Encyclopedia of Genes and Genomes (KEGG, version 2024), and Gene Ontology (GO) databases. Because microsporidian KEGG/GO annotation is sparse, functional annotation was performed by homology to UniProtKB/Swiss-Prot, KEGG, and Gene Ontology using fungal ortholog identifiers—*Saccharomyces cerevisiae* (KEGG sce) and *Schizosaccharomyces pombe* (KEGG spo)—which are phylogenetically more appropriate references for microsporidia than mammalian orthologs.

For functional classification, GO terms were categorized into Biological Process (BP), Molecular Function (MF), and Cellular Component (CC). KEGG pathways were analyzed using Fisher’s exact test against a fungal (*S. cerevisiae*, sce) ortholog background, and statistical significance for enriched pathways was defined by a Fisher’s exact test *P* value < 0.05. Furthermore, protein-protein interaction (PPI) networks were constructed using the STRING database (version 12.0) against the *Saccharomyces cerevisiae* proteome and visualized via Cytoscape software to elucidate functional modules among the DEPs. It should be noted that the PPI network was constructed using *S. cerevisiae* STRING interaction data inferred for *P. locustae* orthologs; as such, it represents a functional connectivity map based on conserved protein interactions and may not capture microsporidia-specific interactions.

### Validation of differential gene expression by qRT-PCR

The transcriptional levels of selected DEPs were validated using quantitative real-time PCR (qRT-PCR). Purified spore aliquots (1 × 10^9^ spores/mL) were washed three times with phosphate-buffered saline. Total RNA was extracted using the TRIzol reagent (Takara Bio, Dalian, China). RNA integrity was assessed by 1% agarose gel electrophoresis, while purity and concentration were determined using a NanoDrop 2000 spectrophotometer (Thermo Fisher Scientific, Waltham, MA, USA), ensuring 260/280 and 260/230 ratios were within optimal ranges. First-strand cDNA was synthesized from 1 μg of total RNA using the PrimeScript RT Reagent Kit (RR047A, Takara Bio, Dalian, China) following the manufacturer’s instructions. qRT-PCR was performed on a Bioer Gene 9600 Real-Time PCR System (Hangzhou Bioer Technology, China) using TB Green Premix Ex Taq (RR820A, Takara Bio, Dalian, China). Each 20 μL reaction contained 1 μL of cDNA as the template, with three biological replicates per treatment. No-template controls and no-reverse-transcriptase controls were included to verify amplification specificity. The cycling conditions consisted of an initial denaturation at 95°C for 30 s, followed by 40 cycles of 94°C for 5 s and 60°C for 30 s. A final melting curve analysis was conducted to confirm primer specificity. The small subunit ribosomal RNA (SSU) gene served as the internal reference, and relative expression levels were calculated using the 2^−(∆∆Ct)^ method. All gene-specific primers are listed in Table 1. Genes for qRT-PCR validation were selected based on (i) significant differential expression (FC ≥ 1.5, *P* < 0.05) and (ii) functional relevance to stress response pathways.

**TABLE 1:** The primer pairs and probe used for fluorescence quantitative PCR^*a*^

Gene	Primer	Sequence (5′−3′)
*P.L.EF*	F	CTGAAACGCTGCACGCAGGT
	R	GGAGGAGTGGGTTCGCAAGAT
*P.L.RIP2*	F	CTCGACCTTTTCCATCTGGGC
	R	ACGGTGCGTCTTCTTTGTGTG
*P.L.MP*	F	AGTGCCATCACATCAATTTTGC
	R	TACCTGGTTCACGCTCTCCTTC
*P.L.PDI*	F	ACGGTGCGTGAAAAACAACGA
	R	CTGAGGGCAACGAAAAGAATG
*P.L.SRP*	F	TTTCCAACACCATTCACACCCA
	R	AGATTTGTGAAAAGTGTGGACC
*P.L.TBCD*	F	CCTCAAGTACAACGGCACACT
	R	TCTCTACAATTCCCGCAAACA
*P.L.GTP*	F	TGATGTAGTATGCGTCGCTTAG
	R	ACGGGTTTCAATTGGAGTTTGA
*P.L.APBA*	F	GCAGAACAAAGCACCCAAAAT
	R	CAGAACGCAAAGCAAAACCAT
*P.L.E2*	F	CGTTTTTCGAGGATGGCACCT
	R	CTGGCATACACATTGGGATGG
*P.L.ZF*	F	TGCTTCGAAACGAAAAGGGAAA
	R	GGTGCAAACAAACACTGCATGA
*P.L.UBL*	F	TGCAAAGGGAAGCCAGACAGA
	R	AGCCAGGAAACATCGAAATCA
*P.L.NSF*	F	TGCTGTATGCGTTTCTTGCGT
	R	GCTTTGCTCTCTCTCCTCCGT
*P.L.KARS*	F	GCGTCCAGGGCTTCCTGAGTA
	R	TCCAGCGGCGAAAGGTTTATT
*P.L.FH*	F	TGGAGATAGTGGGCGATGTGA
	R	CTATCTCCTTTGTGCGCAAGC
*P.L.SSU*	F	TGATCAAGGACGAAGGCCG
	R	CAATTTCTCCCGGACACAGCC

^*a*^ *P.L*., *Paranosema locustae*; EF, elongation factor; MP, membrane protein; RIP2, ribosome-inactivating protein RIP-II; PDI, protein disulfide isomerase (thiol-disulfide isomerase); SRP, signal recognition particle; TBCD, tubulin-specific chaperone D; GTP, GTP-binding nucleolar protein; APBA, amyloid beta A4 precursor protein-binding family protein; E2, ubiquitin-conjugating enzyme E2; ZF, zinc finger protein; UBL, ubiquitin-like protein; NSF, N-ethylmaleimide-sensitive factor; KARS, lysyl-tRNA synthetase; FH, frataxin; SSU rRNA, small subunit ribosomal RNA (internal reference).

## RESULTS

### SDS-PAGE profiles of spore proteins under stress treatments

**Fig 1:**
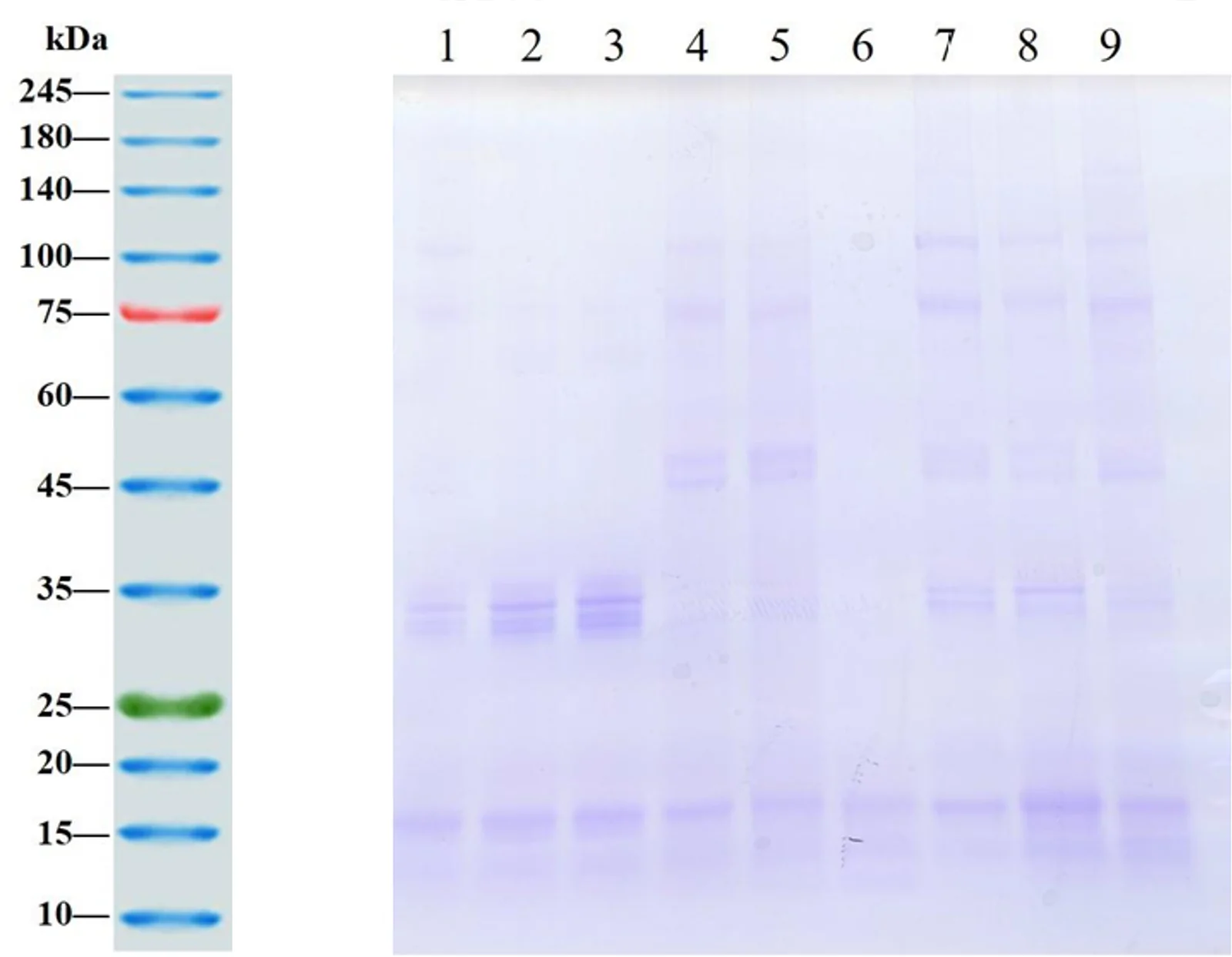
SDS-PAGE profiles of *P. locustae* proteins under different stress conditions. Lanes 1–3: control group (CK, spores stored at −20°C); lanes 4–6: UV group (spores exposed to UV irradiation for 24 h); lanes 7–9: dry group (spores subjected to desiccation at 40°C for 5 days). Each group was analyzed in triplicate. The leftmost lane shows the protein molecular weight marker. Minor variations in band intensity at approximately 75 kDa were observed between treatment groups and the control. Slight variations in total protein loading across individual lanes reflect minor technical inconsistencies during sample preparation and were normalized during subsequent DIA-MS quantification.

To preliminarily evaluate the impact of environmental stress on the proteomic landscape of *P. locustae*, total protein extracts from the control (CK), UV-irradiated, and desiccated groups were analyzed via SDS-PAGE. Distinct variations in protein expression patterns were observed across the different treatment groups. Compared to the control, both the UV-irradiated and desiccated groups exhibited a noticeable increase in protein abundance at approximately 75 kDa (Fig. 1).

Furthermore, stress-specific alterations were identified: the UV-irradiated group showed a marked reduction in protein intensity near 36 kDa, whereas the desiccated group displayed a prominent, high-level expression band at 100 kDa (Fig. 1). It should be noted that the total protein loading in lane 6 (UV group, replicate 3) appeared slightly lower than that in lanes 4–5 based on SDS-PAGE visualization. However, this variation was attributable to minor pipetting inconsistency during sample preparation and did not affect the subsequent DIA-based quantitative proteomic analysis, as DIA-MS employs peptide-level label-free quantification with internal normalization across all samples (Fig. S1). These discernible shifts in protein profiles provide initial evidence of a specialized stress-response mechanism in *P. locustae* spores, warranting further systematic investigation via DIA-based quantitative proteomics.

### Quantitative proteomic profiling and DEP identification

A total of 863 unique peptides and 187 high-confidence proteins were identified and quantified across all spore samples via DIA-based proteomics (Table 2; Table S1). To characterize the proteomic response of *P. locustae* to environmental challenges, we performed differential expression analysis between the stress-treated groups and the control (CK).

**TABLE 2:** Statistics of DIA protein identification results

Group	Sample name	Number of protein groups	Unique peptides
CK	CK1	171	728
	CK2	167	671
	CK3	165	635
	CK-total	182	823
Dry	Dry1	171	746
	Dry2	173	745
	Dry3	178	762
	Dry-total	184	821
UV	UV1	178	757
	UV2	180	771
	UV3	176	668
	UV-total	186	827
Total		187	863

**Fig 2:**
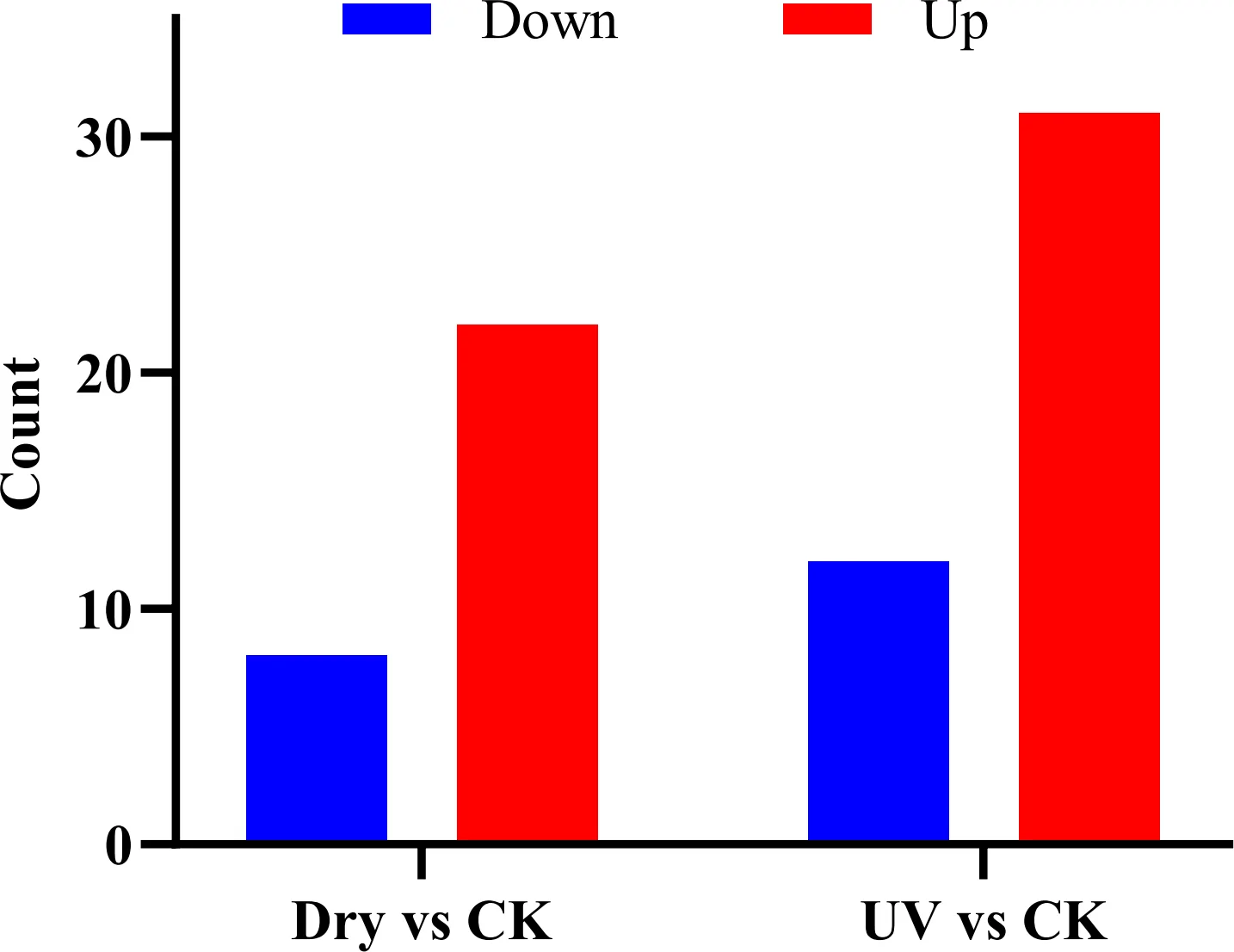
Number of DEPs under desiccation and UV stress. Bar chart summarizing DEP counts for each treatment.

Under desiccation stress (40°C), 30 proteins were identified as DEPs, consisting of 22 upregulated and 8 downregulated proteins. In response to UV irradiation, a more extensive proteomic shift was observed, with 43 DEPs identified, including 31 upregulated and 12 downregulated proteins (Fig. 2). These results indicate that both desiccation and UV irradiation trigger significant proteomic remodeling in *P. locustae* spores, with UV stress eliciting a broader quantitative response in the identified proteome.

To verify the taxonomic assignment of these DEPs, all 73 proteins (57 unique accessions after deduplication) were subjected to BLASTp searches against the NCBI nr database (E-value < 1e^−5^, identity > 30%, coverage > 50%), which confirmed their microsporidian origin (Table S2). Notably, the reanalysis using the custom *A. locustae* database captured not only conserved housekeeping proteins but also microsporidia-specific proteins, including polar tube proteins (PTP2, PTP2b, PTP2c), spore wall protein SWP1, and sporoplasm surface proteins, indicating that the customized database approach successfully identified both core and spore-specific proteomic features.

Of note, the microsporidia-specific structural and invasion-related proteins that were confidently detected in the dormant spores—the polar tube proteins PTP2, PTP2b, and PTP2c, the spore wall protein SWP1, and sporoplasm surface protein 1—did not reach statistical significance (*P* > 0.05) under either desiccation or UV-C irradiation, although some showed non-significant trends (e.g., SWP1 tended to increase under UV-C). This is consistent with their biological function: these proteins are pre-assembled, constitutive components of the spore invasion apparatus that are synthesized during sporulation and stored in the mature dormant spore, and their abundance is therefore not expected to be remodeled by short-term environmental stress. In contrast, the proteins that responded to stress belonged predominantly to housekeeping and proteostasis-related categories, including molecular chaperones, proteasome subunits, and translation factors, indicating that the dormant spore responds to desiccation and UV-C exposure by mobilizing a limited, proteostasis-centered stress program rather than by remodeling its structural and invasion-related machinery.

### GO functional classification of DEPs under desiccation and UV stress

**Fig 3:**
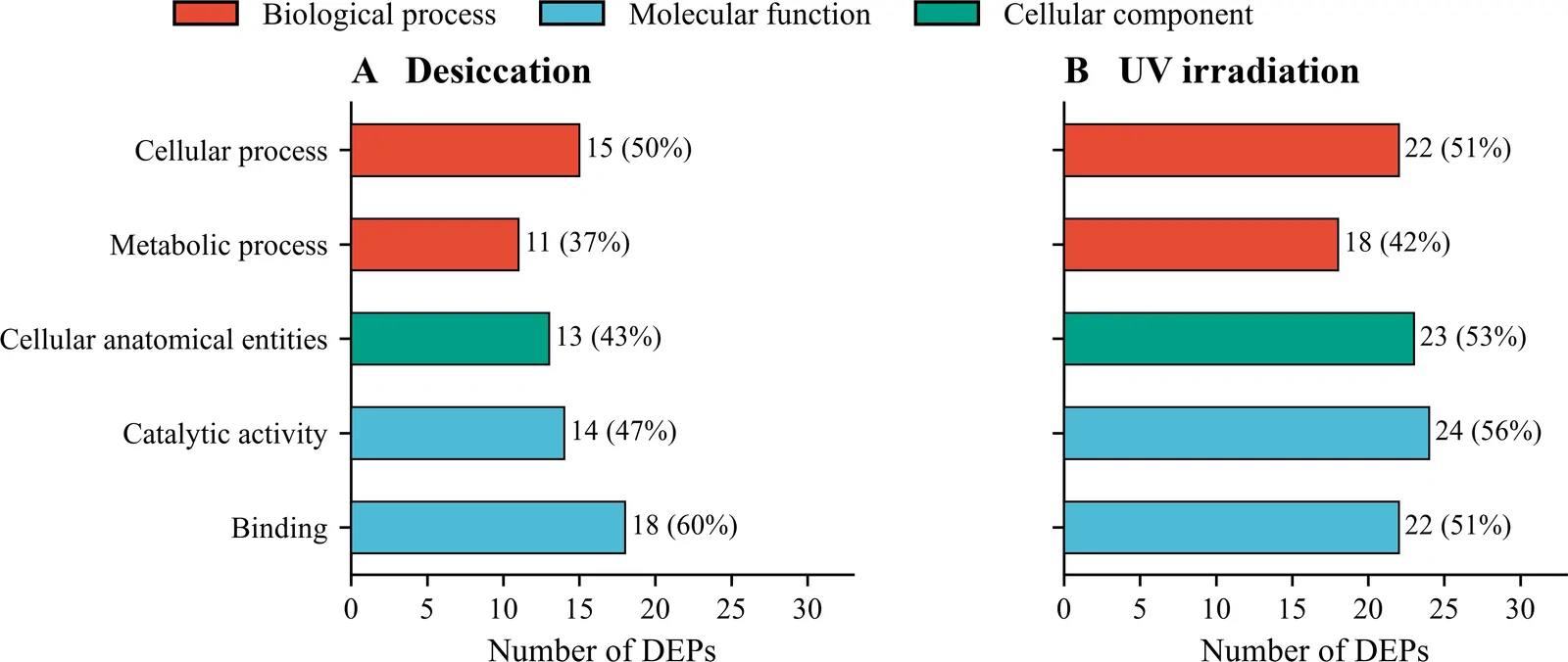
GO functional classification of the *P. locustae* proteome under stress. Note: (**A**) desiccation-treated group; (**B**) UV-irradiated group. All identified proteins were categorized using GO Level 2 annotation. The vertical axis represents the specific Level 2 classification terms. The horizontal axis indicates the protein count, and the value label next to each bar displays the count and the relative percentage (number of proteins in a term/total annotated proteins). The specific number of proteins and the corresponding percentage are labeled next to each bar.

To further elucidate the functional characteristics of the identified proteome, GO classification was performed, categorizing proteins into BP, MF, and CC. The analysis revealed that the proteomic response in both the desiccation- and UV-treated groups was predominantly centered on metabolic reconfiguration and catalytic modulation (Fig. 3).

In the BP category, “cellular process” (desiccation: 15 proteins; UV: 22 proteins) and “metabolic process” (desiccation: 11 proteins; UV: 18 proteins) emerged as the most represented terms. Regarding CC, the majority of DEPs were localized to “cellular anatomical entities,” with 13 proteins identified in the desiccation group compared to 23 in the UV group.

In terms of MF, the response was highly concentrated in “catalytic activity” (desiccation: 14 proteins; UV: 24 proteins) and “binding” (desiccation: 18 proteins; UV: 22 proteins). These results suggest that while both stressors activate similar functional modules, UV irradiation induces a more extensive recruitment of the functional proteome to counteract environmental damage.

### KEGG pathway mapping and enrichment analysis

**Fig 4:**
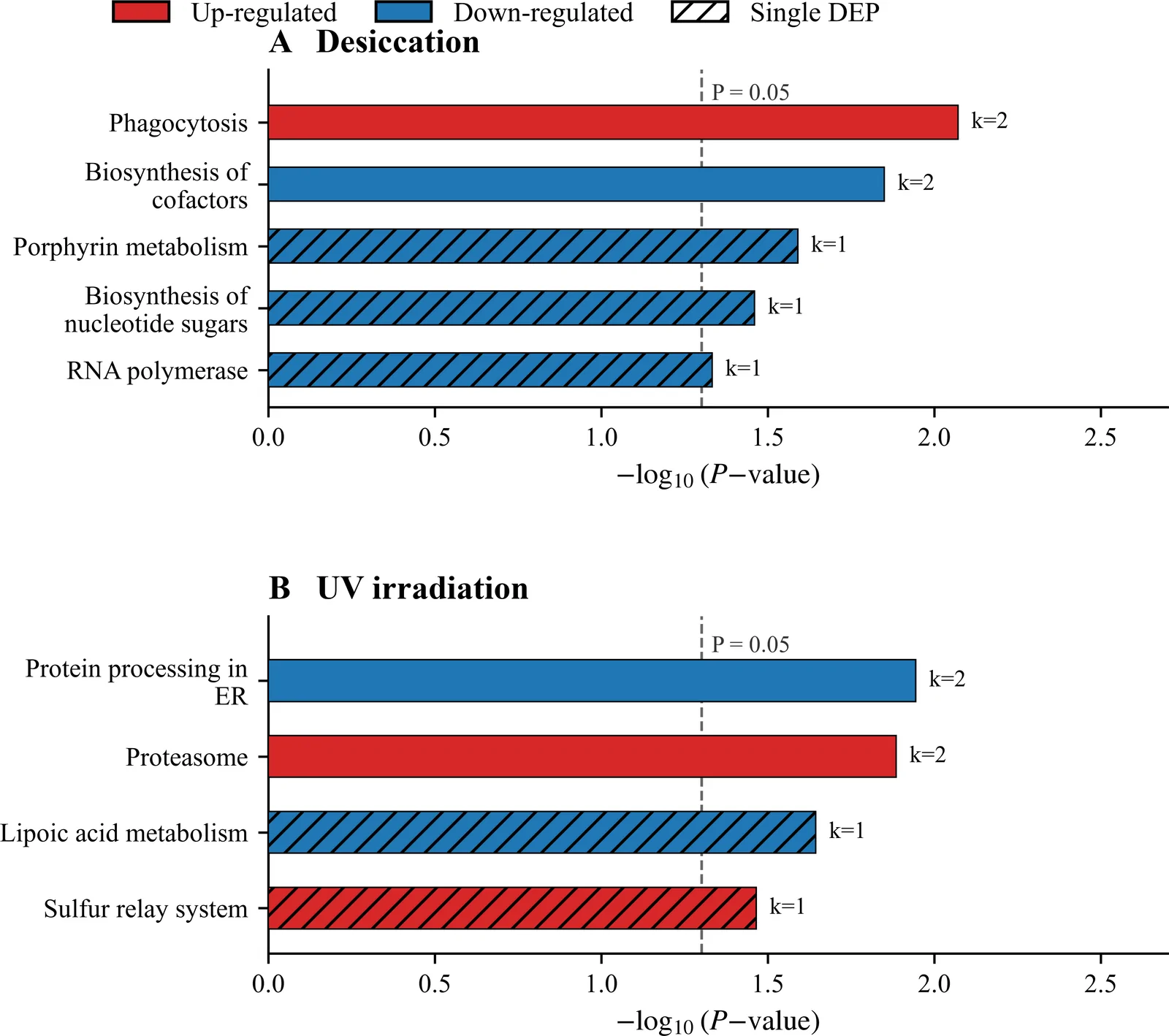
KEGG pathway enrichment analysis of DEPs in *P. locustae* under desiccation and UV stress. Note: (**A**) desiccation-treated group; (**B**) UV-irradiated group. The horizontal axis represents the degree of statistical significance (−log_10_ *P*). Bar color indicates the direction of regulation: red, pathways enriched among upregulated DEPs; blue, pathways enriched among downregulated DEPs. Only pathways with a Fisher’s exact test *P* < 0.05 are shown; the dashed vertical line marks *P* = 0.05. Solid bars indicate pathways with ≥2 DEPs; hatched bars indicate single-DEP pathways treated as exploratory. The number of DEPs (k) is labeled at the end of each bar.

To identify the specific metabolic and signaling pathways modulated by environmental stress, we mapped the DEPs to the KEGG database using fungal orthologs—*Saccharomyces cerevisiae* (KEGG sce) and *Schizosaccharomyces pombe* (KEGG spo)—which are phylogenetically more appropriate references for microsporidia than mammalian orthologs. Because microsporidian proteomes are highly reduced and divergent, a subset of DEPs could not be assigned a fungal ortholog with pathway annotation. In the desiccation group (dry vs CK), 11 of the 30 DEPs were mapped to *S. cerevisiae* orthologs with pathway annotation (12 of 30 to *S. pombe*). Enrichment analysis (Fisher’s exact test, *P* < 0.05) identified the phagosome-related pathway (KEGG sce04145/spo04145, “Phagocytosis”), which in fungi is represented by vacuolar/endosomal trafficking components, as the primary enriched pathway, driven by the vacuolar H^+^-ATPase subunit (EED44510.1) and β-tubulin (EED41498.1) (Table 3; Fig. 4A). Direction-stratified analysis of the downregulated subset revealed significant enrichment of biosynthetic pathways, including biosynthesis of cofactors, nucleotide sugar metabolism, porphyrin metabolism, and RNA polymerase (*P* < 0.05, Table 3), consistent with the suppression of basal metabolism under extreme desiccation.

**TABLE 3:** Significant differences in protein content were observed between the dry group and the control group^*a*^

Pathway	No. of DEPs	Accession	Protein name	*P* value	FC
Phagocytosis (sce04145/spo04145)	2	EED44510.1	Vacuolar H^+^-ATPase subunit d (VMA6)	0.033	2.292 +
		EED41498.1	β-tubulin (TUB2)	0.050	1.776 +
Aminoacyl-tRNA biosynthesis (sce00970)	2	AAB62548.1	Glutaminyl-tRNA synthetase (GLN4)	0.016	2.296 +
		EDQ31175.1	Glutamyl-tRNA synthetase (GUS1)	0.049	0.425 −
Biosynthesis of cofactors (sce01240)	2	AAT12356.1	Mannose-1-phosphate guanylyltransferase (MPG1/PSA1)	0.027	0.435 −
		EDQ31175.1	Glutamyl-tRNA synthetase (GUS1)	0.049	0.425 −
Protein processing in endoplasmic reticulum (sce04141)^*b*^	1	EED43833.1	Protein disulfide isomerase (PDI1)	0.014	0.220 −
Peroxisome (sce04146)^*b*^	1	AAY27408.1	Manganese superoxide dismutase (SOD2)	0.021	1.868 +
Proteasome (sce03050)^*b*^	1	EED44392.1	26S proteasome regulatory subunit RPT2	0.038	2.567 +
RNA polymerase (sce03020)^*b*^	1	AAD12605.1	RNA polymerase II largest subunit (RPO21)	0.043	0.635 −
Nucleotide excision repair (sce03420)^*b*^	1	AAD12605.1	RNA polymerase II largest subunit (RPO21)	0.043	0.635 −
Nucleocytoplasmic transport (sce03013)^*b*^	1	AAT12341.1	GTP-binding nucleolar protein (GSP1/Ran)	0.029	1.967 +
RNA degradation (sce03018)^*b*^	1	EED44154.1	Exoribonuclease II (DIS3)	0.031	1.842 +

^*a*^ DEPs with significant differential expression between the dry-treated group and the control group are listed based on fungal ortholog mapping (*Saccharomyces cerevisiae*, KEGG sce; *Schizosaccharomyces pombe*, KEGG spo). “No. of DEPs” indicates the number of differentially expressed proteins mapped to each pathway.

^*b*^ Pathway represented by a single DEP; its enrichment is driven by one protein rather than coordinated pathway-level regulation and is therefore treated as exploratory and excluded from the biological interpretation. The ratio of fold change (FC) represents protein abundance in the dry-treated group relative to the control; “+” indicates upregulation (FC ≥ 1.5) and “−” indicates downregulation (FC ≤ 0.67). AAD12605.1 (RNA polymerase II largest subunit) additionally mapped to Nucleotide excision repair (sce03420/spo03420); AAT12341.1 (GTP-binding nucleolar protein) additionally mapped to Ribosome biogenesis in eukaryotes (sce03008/spo03008) and Viral life cycle (sce03250/spo03250); EED44510.1 additionally mapped to Lysosome biogenesis (sce04142/spo04142) and Oxidative phosphorylation (sce00190/spo00190); EED41498.1 additionally mapped to Motor proteins (sce04814/spo04814).

Because the confidently identified proteome was limited to 187 protein groups, many KEGG pathways were represented by only a single DEP. Although the Fisher’s exact test *P* values are statistically valid against this background, a pathway enrichment based on a single DEP does not constitute evidence of coordinated pathway-level regulation and was therefore treated as exploratory; moreover, because the identified proteome is biased toward abundant, housekeeping proteins rather than a random sample of the proteome, pathway over-representation may partly reflect detection bias. Accordingly, in Tables 3 and 4 we report the number of DEPs per pathway and flag single-DEP pathways (*); the biological interpretation below is restricted to pathways represented by two or more DEPs—under desiccation, vacuolar/endosomal trafficking (two DEPs); under UV irradiation, protein processing in the endoplasmic reticulum (three DEPs), the proteasome (two DEPs), and the integrated stress response (two DEPs).

**TABLE 4:** Significant differences in protein content were observed between the UV-exposed group and the control group^*a*^

Pathway	No. of DEPs	Accession	Protein name	*P* value	FC
Protein processing in endoplasmic reticulum (sce04141/spo04141)	3	AAT12327.1	Ubiquilin (DSK2)	0.016	1.522 +
		EED43833.1	Protein disulfide isomerase (PDI1)	0.020	0.321 −
		EED44519.1	Hsp70 chaperone BiP (KAR2)	0.017	0.439 −
Proteasome (sce03050)	2	AAT12385.1	26S proteasome regulatory subunit RPT1	0.013	1.757 +
		EDQ31099.1	19S proteasome regulatory subunit RPT6	0.026	1.827 +
Integrated stress response (ISR) signaling pathway (sce04156/spo04156)	2	EED44519.1	Hsp70 chaperone BiP (KAR2)	0.017	0.439 −
		EDQ31180.1	Serine/threonine-protein phosphatase PP1 (GLC7)	0.045	1.715 +
Aminoacyl-tRNA biosynthesis (sce00970)	3	EDQ31218.1	Lysyl-tRNA synthetase (KRS1)	0.010	4.541 +
		AAB62548.1	Glutaminyl-tRNA synthetase (GLN4)	0.026	3.071 +
		AAC41564.1	Isoleucyl-tRNA synthetase (ILS1)	0.048	1.724 +
Sulfur relay system (sce04122)^*b*^	1	AAY27413.1	Cysteine desulfurase (NFS1)	0.015	2.301 +
Protein export (sce03060)^*b*^	1	EED44519.1	Hsp70 chaperone BiP (KAR2)	0.017	0.439 −
Thiamine metabolism (sce00730)^*b*^	1	AAY27413.1	Cysteine desulfurase (NFS1)	0.015	2.301 +
Basal transcription factors (sce03022)^*b*^	1	EED43723.1	TATA-binding protein-associated factor (TAF)	0.044	0.098 −
Nucleocytoplasmic transport (sce03013)^*b*^	1	AAT12341.1	GTP-binding nucleolar protein (GSP1/Ran)	0.006	2.286 +
Endocytosis (sce04144)^*b*^	1	AAT12346.1	GTPase-activating protein	0.013	1.880 +
Autophagy (sce04138)^*b*^	1	ACI87875.1	Autophagy-related protein	0.014	2.030 +
Phagocytosis (sce04145)^*b*^	1	EED41498.1	β-tubulin (TUB2)	0.002	2.598 +
Porphyrin metabolism (sce00860)^*b*^	1	AAY27415.1	Frataxin (YFH1)	0.044	1.731 +
Lipoic acid metabolism (sce00785)^*b*^	1	AAL28054.1	Pyruvate dehydrogenase E1 component	0.030	0.627 −
Cell cycle (sce04111)^*b*^	1	EED44274.1	Condensin subunit SMC4	0.020	0.544 −

^*a*^ DEPs with significant differential expression between the UV-treated group and the control group are listed based on fungal ortholog mapping (*Saccharomyces cerevisiae*, KEGG sce; *Schizosaccharomyces pombe*, KEGG spo). “No. of DEPs” indicates the number of differentially expressed proteins mapped to each pathway.

^*b*^ Pathway represented by a single DEP; its enrichment is driven by one protein rather than coordinated pathway-level regulation and is therefore treated as exploratory and excluded from the biological interpretation. The ratio of fold change (FC) represents protein abundance in the UV-treated group relative to the control; “+” indicates upregulation (FC ≥ 1.5) and “−” indicates downregulation (FC ≤ 0.67). EED44519.1 (molecular chaperone) additionally mapped to Protein export (sce03060/spo03060) and the integrated stress response (sce04156/spo04156); AAY27413.1 (cysteine desulphurase) additionally mapped to Thiamine metabolism (sce00730/spo00730); AAT12341.1 (GTP-binding nucleolar protein) additionally mapped to Ribosome biogenesis in eukaryotes (sce03008/spo03008) and Viral life cycle (sce03250/spo03250); EED41498.1 additionally mapped to Motor proteins (sce04814/spo04814); AAL28054.1 additionally mapped to the citrate cycle (sce00020), glycolysis (sce00010), and pyruvate metabolism (sce00620).

**Fig 5:**
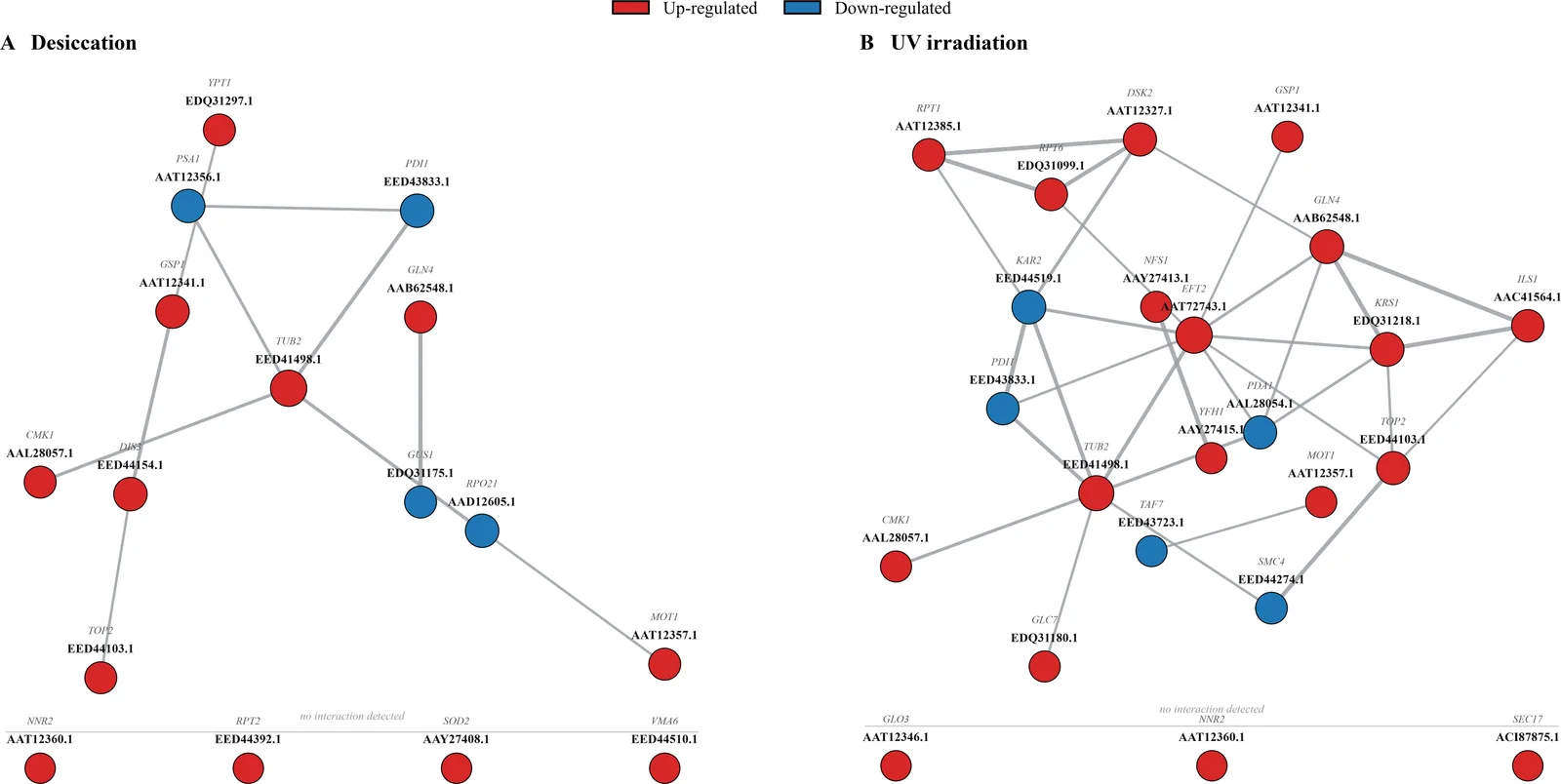
PPI networks of DEPs in *P. locustae* spores (*S. cerevisiae* orthologs). Note: (**A**) desiccation-treated group; (**B**) UV-irradiated group. PPI networks were constructed using the STRING database (v12.0) against the *Saccharomyces cerevisiae* proteome and visualized with Cytoscape. Nodes represent *P. locustae* DEPs mapped to their *S. cerevisiae* orthologs, colored by the direction of regulation (red, upregulated; blue, downregulated); node size is proportional to the number of interactions (degree). Edges represent *S. cerevisiae* interactions with a combined score ≥0.4. DEPs lacking medium-confidence interactions are shown below the horizontal line.

In the UV-irradiated group (UV vs CK), 18 of the 43 DEPs were mapped to *S. cerevisiae* orthologs with pathway annotation (18 of 43 to *S. pombe*). Direction-stratified enrichment analysis (Fisher’s exact test, *P* < 0.05) identified protein processing in the endoplasmic reticulum (two downregulated DEPs: the chaperone BiP/KAR2 and the protein disulfide isomerase PDI1) and the proteasome (two upregulated DEPs: the 19S regulatory subunits RPT1 and RPT6) as significantly enriched (Table 4; Fig. 4B). Beyond these enriched pathways, a coordinated ER-proteostasis module was evident: the ubiquilin DSK2 was upregulated in parallel with the downregulation of BiP/KAR2 and PDI1, and the integrated stress response (ISR) components changed in opposite directions (BiP/KAR2 down; the phosphatase PP1/GLC7 up) (Fig. 5B). Three aminoacyl-tRNA synthetases (KRS1, GLN4, ILS1) were also coordinately upregulated, although this large pathway did not reach statistical over-representation (Table 4). Together, these changes are consistent with an ER-proteostasis-centered response to UV-induced proteotoxic stress.

Further analysis of individual DEPs provided specific molecular insights. In the desiccation group, the vacuolar/endosomal trafficking pathway was driven by upregulation of the vacuolar H^+^-ATPase subunit (EED44510.1) and β-tubulin (EED41498.1) (Table 3). In parallel, key proteins driving basal biosynthetic and genetic processes were suppressed, notably RNA polymerase II (AAD12605.1; mapped to the fungal RNA polymerase and nucleotide excision repair pathways) and glutamyl-tRNA synthetase (EDQ31175.1; mapped to aminoacyl-tRNA biosynthesis), consistent with a selective downregulation of biosynthetic metabolism rather than a generalized protein shutdown.

In the UV-irradiated group, the protein processing in the endoplasmic reticulum pathway showed a mixed response: the protein disulfide isomerase PDI (EED43833.1) and the molecular chaperone BiP/KAR2 (EED44519.1) were significantly downregulated, whereas the ubiquilin homolog DSK2 (AAT12327.1) was upregulated (Table 4). The downregulation of the two endoplasmic-reticulum-resident folding components (PDI and BiP) indicates that prolonged UV exposure may compromise the protein-folding capacity of the endoplasmic reticulum in *P. locustae* spores, despite the concurrent upregulation of the proteasome and other stress-responsive elements.

### Protein-protein interaction network analysis

To further elucidate the functional coordination among the identified DEPs, PPI networks were constructed using the STRING database and visualized via Cytoscape. It should be noted that this network is derived from *S. cerevisiae* interaction data mapped onto *P. locustae* orthologs; it represents a functional connectivity map of conserved interactions.

In the desiccation group, the upregulated DEPs formed a sparse network centered on the cytoskeletal protein β-tubulin (TUB2), with a second cluster linking the GTPases GSP1 and YPT1 to the exosome subunit DIS3 and topoisomerase II (TOP2), implicating cytoskeletal remodeling, trafficking, and RNA metabolism in the desiccation response (Fig. 5A; Table 5).

**TABLE 5:** Differentially expressed proteins of *P. locustae* and their *S. cerevisiae* orthologs^*a*^

Accession	*S. cerevisiae* ortholog	Protein function	Desiccation	UV irradiation
AAL28057.1	*CMK1*	Calmodulin-dependent protein kinase	Up	Up
EED44154.1	*DIS3*	Exosome subunit DIS3	Up	–
AAT12327.1	*DSK2*	Ubiquilin (UBL-UBA protein)	–	Up
AAT72743.1	*EFT2*	Translation elongation factor 2	–	Up
EDQ31180.1	*GLC7*	Ser/Thr protein phosphatase PP1	–	Up
AAB62548.1	*GLN4*	Glutaminyl-tRNA synthetase	Up	Up
AAT12346.1	*GLO3*	ADP-ribosylation factor GTPase-activating protein	–	Up
AAT12341.1	*GSP1*	GTP-binding nucleolar protein (Ran)	Up	Up
EDQ31175.1	*GUS1*	Glutamyl-tRNA synthetase	Down	–
AAC41564.1	*ILS1*	Isoleucyl-tRNA synthetase	–	Up
EED44519.1	*KAR2*	Hsp70 chaperone BiP	–	Down
EDQ31218.1	*KRS1*	Lysyl-tRNA synthetase	–	Up
AAT12357.1	*MOT1*	DNA-binding ATPase (chromatin remodeler)	Up	Up
AAY27413.1	*NFS1*	Cysteine desulfurase	–	Up
AAT12360.1	*NNR2*	NADHX dehydratase	Up	Up
AAL28054.1	*PDA1*	Pyruvate dehydrogenase E1 α subunit	–	Down
EED43833.1	*PDI1*	Protein disulfide isomerase	Down	Down
AAT12356.1	*PSA1*	Mannose-1-phosphate guanylyltransferase	Down	–
AAD12605.1	*RPO21*	RNA polymerase II largest subunit	Down	–
AAT12385.1	*RPT1*	26S proteasome regulatory subunit Rpt1	–	Up
EED44392.1	*RPT2*	26S proteasome regulatory subunit Rpt2	Up	–
EDQ31099.1	*RPT6*	19S proteasome regulatory subunit Rpt6	–	Up
ACI87875.1	*SEC17*	α-SNAP (Sec17, vesicular trafficking)	–	Up
EED44274.1	*SMC4*	Condensin subunit SMC4	–	Down
AAY27408.1	*SOD2*	Manganese superoxide dismutase	Up	–
EED43723.1	*TAF7*	TATA-binding protein-associated factor	–	Down
EED44103.1	*TOP2*	DNA topoisomerase II	Up	Up
EED41498.1	*TUB2*	β-tubulin	Up	Up
EED44510.1	*VMA6*	Vacuolar H^+^-ATPase subunit d	Up	–
AAY27415.1	*YFH1*	Frataxin	–	Up
EDQ31297.1	*YPT1*	Rab family GTPase (vesicular transport)	Up	–

^*a*^ DEPs: fold change ≥1.5 (up) or ≤0.67 (down), *P* < 0.05. Up, upregulated; Down, downregulated; –, not differentially expressed in that treatment.

The downregulated DEPs, including protein disulfide isomerase (PDI1), the guanylyltransferase PSA1, and RNA polymerase II (RPO21), are associated with ER quality control and biosynthetic/transcriptional functions, consistent with a global downregulation of anabolism in desiccated spores (Fig. 5A; Table 5).

For the UV-irradiated group, the PPI network was markedly denser and revealed two coordinated upregulated clusters: the translation machinery (elongation factor 2, EFT2, together with the lysyl-, glutaminyl-, and isoleucyl-tRNA synthetases KRS1, GLN4, and ILS1) and the 26S proteasome regulatory subunits RPT1 and RPT6 together with the ubiquilin DSK2. The ER chaperone BiP (KAR2) and protein disulfide isomerase (PDI1) were downregulated, underscoring a coordinated proteostatic response in which protein synthesis and degradation machinery compensate for UV-induced damage to the endoplasmic reticulum (Fig. 5B; Table 5).

### Validation of DEP transcriptional levels via qRT-PCR

**Fig 6:**
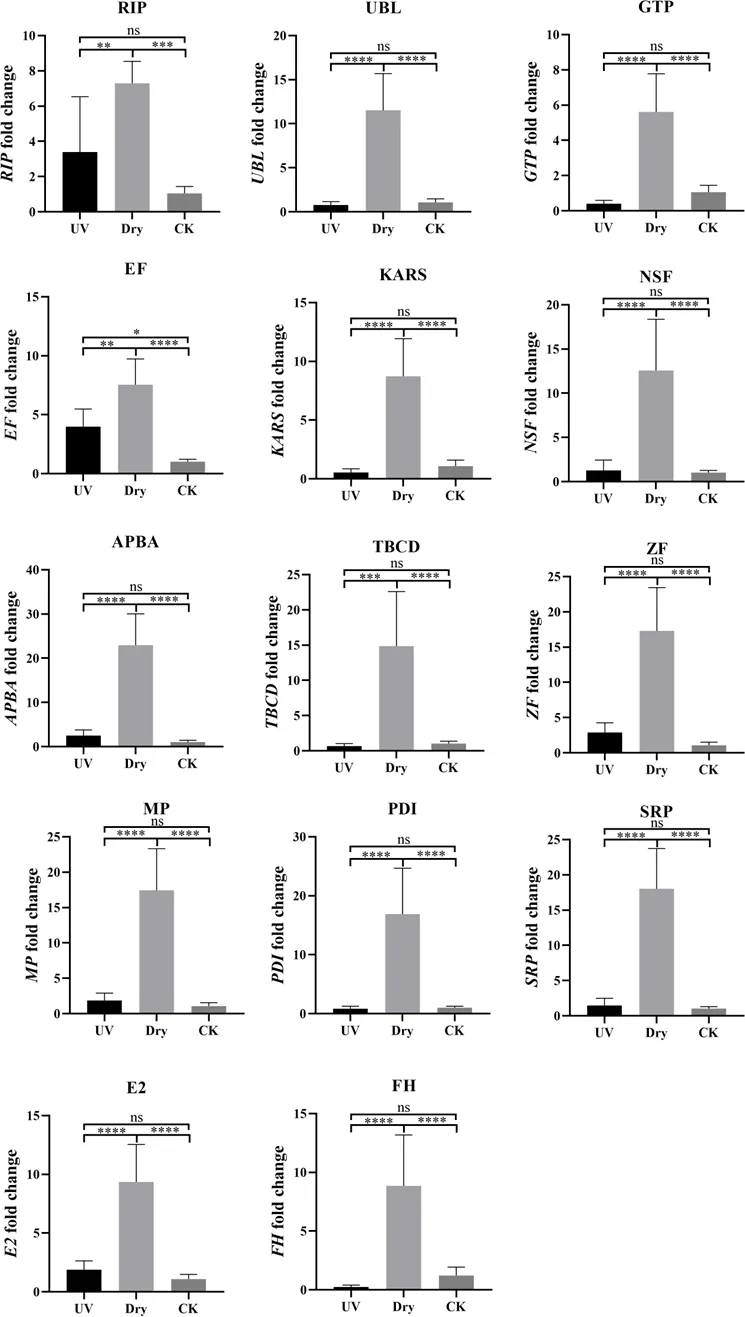
Validation of selected DEP expression at the transcriptional level. Note: relative mRNA expression levels of *P. locustae* genes were determined via qRT-PCR under desiccation and UV irradiation. The SSU gene was used as an internal control for normalization. Data are presented as the mean ± SD (*n* = 3 biological replicates). Asterisks indicate statistically significant differences compared to the control group (*P < 0.05, **P < 0.01, ***P < 0.001, **P < 0.0001), and 'ns' indicates no significant difference (*P* > 0.05).

To verify the correlation between protein abundance and their corresponding mRNA levels, qRT-PCR was performed on selected DEPs identified from the *P. locustae* proteome. Complete results for all 14 tested genes under both desiccation and UV conditions are provided in Table S3. Following 5 days of desiccation treatment, 9 of the 14 tested genes showed significant upregulation at the transcriptional level, including ribosome-inactivating protein (RIP-II), GTP-binding nucleolar protein, and amyloid beta A4 precursor protein-binding family protein (all *P* < 0.05, Fig. 6). These results demonstrate a consistent trend between the transcriptome and proteome in response to desiccation stress.

In contrast, the UV-irradiated group exhibited a distinct regulatory pattern. After 24 h of UV exposure, only 3 of 14 tested genes showed significant transcriptional changes relative to the control, despite their observed changes at the protein level. A notable exception was the translation elongation factor EF, which displayed a significant increase in mRNA expression (Fig. 6). This divergence suggests that while the transcriptional response to UV stress is relatively limited, *P. locustae* may rely more heavily on post-transcriptional modifications or translational regulation to modulate its proteomic landscape under radiation.

The limited transcriptional response to UV (3/14 genes) despite broad proteomic changes could reflect three non-mutually-exclusive mechanisms: (i) translational and post-transcriptional regulation plays a dominant role in the UV response, consistent with the upregulation of translation and proteostasis machinery; (ii) the observed protein changes partly reflect altered protein stability—e.g., ubiquitin-proteasome-system-dependent differential degradation—rather than *de novo* synthesis; and (iii) the 24-h sampling point may have missed an earlier transcriptional peak. Distinguishing among these will require time-resolved transcriptomic and translational profiling and targeted protein-stability assays.

## DISCUSSION

Environmental fluctuations pose a significant challenge to the biocontrol efficacy of the microsporidian parasite *Paranosema locustae*. Our previous work, conducted on the same spore stock and under identical treatment conditions, demonstrated that *P. locustae* spores retain infectivity following thermal desiccation (40°C) and UV irradiation, a resilience associated with the upregulation of the dormancy factor Lso2 and stress-specific alterations in protein phosphorylation profiles (6, 9). Building on these findings, the present study employed DIA-based quantitative proteomics to systematically characterize the global proteomic responses of *P. locustae* spores to desiccation and UV stress. By integrating differentially expressed proteins with functional pathway analyses, our results reveal that *P. locustae* deploys distinct, stress-dependent molecular responses to cope with contrasting environmental challenges.

Phylogenetic analyses indicate that *Paranosema locustae*, together with members of the phylum Cryptomycota, occupies a basal position or forms an early-diverging sister group within the fungal kingdom (10). This evolutionary placement raises the possibility that *P. locustae* retains regulatory features conserved from ancestral fungal lineages. In fungi, environment-specific transcriptional programs are known to modify the molecular composition of conidia—including stored mRNAs, proteins, and secondary metabolites—and thereby influence gene expression dynamics upon dormancy break (1). Such transcriptional states have been shown to shape post-germination adaptability, including stress tolerance, antifungal drug resistance, and virulence (11).

Consistent with our qRT-PCR validation, type II ribosome-inactivating protein (RIP-II) was significantly upregulated at the transcriptional level under desiccation (see section “Validation of DEP transcriptional levels via qRT-PCR”). Notably, this transcriptional upregulation of RIP-II during desiccation may reflect a preparatory or regulatory role in translational control during dormancy entry. Although RIP-II is classically associated with ribosome inactivation through cleavage of 28S rRNA, its precise role in *Paranosema locustae* remains to be fully elucidated. In parallel, our data suggest a stage-dependent regulation of the transcriptional machinery during desiccation. During early dehydration, spores appear to rely on pre-existing active RNA polymerase II (RNAP II) to support continued transcription and the accumulation of substantial mRNA pools. As desiccation progresses, the observed decline in RNAP II abundance coincides with transcriptional suppression, consistent with the physiological transition toward deep dormancy.

The downregulation of thiol-disulfide isomerase (PDI, Table 4) under UV stress suggests a shift away from costly protein quality control to prioritize immediate survival needs. This aligns with *P. locustae*’s genomic reduction and parasitic lifestyle (12), favoring energy conservation over active repair in harsh environments, a strategy common in microsporidia (13). The transcriptional upregulation of ribosome-inactivating protein (RIP-II, see section “Validation of DEP transcriptional levels via qRT-PCR”) under desiccation may represent a distinct regulatory mechanism for translational control during dormancy entry.

Together, these findings point to a coordinated regulatory program in *P. locustae* that temporally separates transcriptional changes from translational execution. This pattern may reflect an evolutionarily derived survival mechanism in which ancestral features—such as the reliance on pre-stored transcripts (14, 15)—are retained, while being integrated into a regulatory framework characterized by active transcription during desiccation, followed by rapid translation upon host invasion.

Under desiccation treatment, proteins associated with the phagosome-related pathway and transmembrane transport were significantly upregulated in *Paranosema locustae* (Fig. 4A; Table 3). Although these proteins were identified from the parasite rather than the host, their enrichment suggests a potential involvement of *P. locustae* in processes related to host cell entry and intracellular trafficking. Such changes may be functionally relevant to the efficiency of parasitism, thereby providing molecular clues to the distinctive invasion strategy of microsporidia. Microsporidia invade host cells primarily through two routes: active invasion and phagocytosis-mediated entry (16). During the latter process, the polar tube penetrates the host cell membrane to establish a protected invasion synapse, through which the sporoplasm is delivered into the host cell, leading to the formation of a parasitic vacuole that enables evasion of lysosomal degradation (16, 17). This invasion mode allows microsporidia to bypass mature phagosomes and directly access the host intracellular environment, thereby facilitating proliferation (18).

In a broader biological context, efficient transmembrane transport and protein export systems are widely recognized as critical determinants of host-pathogen interactions. For instance, in gram-negative bacteria, protein export is essential for the deployment of virulence factors, nutrient acquisition, and organelle biogenesis (19). Although microsporidia are evolutionarily distinct from bacteria, this comparison highlights a common principle whereby enhanced transport-related processes may support successful host exploitation across diverse microbial pathogens.

The regulatory network involving GTP-binding nucleolar proteins within the nucleocytoplasmic transport pathway may contribute to host interaction-related processes (Tables 3 and 4), potentially influencing parasite-host communication, metabolic coordination, and the deployment of virulence-associated factors. During dormancy, microsporidia exhibit a markedly reduced metabolic rate; however, basal cellular damage, such as oxidative stress-induced impairment of mitosomes and the endoplasmic reticulum, can continue to accumulate. The upregulation of the Rab GTPase YPT1 is consistent with its known role in ER-to-Golgi vesicular transport, which may contribute to protein homeostasis during stress.

Consistent with this interpretation, the observed downregulation of biosynthetic and cofactor metabolism may further contribute to metabolic reallocation and redox homeostasis by limiting spontaneous reactive oxygen species (ROS) generation. Suppression of ROS production has been widely associated with extended spore longevity and enhanced environmental tolerance (9), supporting a physiological framework in which metabolic restraint and targeted quality control jointly promote long-term survival under harsh conditions.

When microsporidia persist as environmental spores, they are unable to acquire exogenous nutrients and must rely exclusively on endogenous energy reserves to sustain ATP production and stress responses (20). Under acute stress conditions, proteins can also be mobilized as an alternative energy source, with cells coordinating translational inhibition and macromolecular turnover through the proteasome and autophagy pathways to rebalance metabolic demands (21, 22). Consistent with this constraint, our proteomic data revealed that UV irradiation induced a coordinated upregulation of proteins associated with the ubiquitin-proteasome system (UPS) and protein processing in the endoplasmic reticulum, alongside increased abundance of components involved in sulfur relay metabolism and the ISR. In eukaryotes, the UPS and autophagy constitute the principal protein degradation systems that maintain proteostasis under stress (23). These conserved paradigms provide a functional framework for interpreting the concurrent activation of protein turnover and translational control observed in *P. locustae* under UV stress.

Mechanistically, *P. locustae* appears to counter UV-induced damage through an energy-rebalancing response. On the one hand, activation of the UPS facilitates the removal of misfolded or oxidatively damaged proteins (Fig. 4B; Table 4), while the sulfur relay pathway (KEGG sce04122/spo04122, involved in tRNA thiolation) and increased DNA topoisomerase II abundance support repair processes. This multilayered oxidative stress response mirrors conserved regulatory logics in fungi. For instance, in the white-rot fungus *Pleurotus ostreatus*, the transcription factor Rim101 coordinates environmental pH sensing and oxidative stress defense (24). Although microsporidia possess highly reduced genomes and lack many canonical transcriptional regulators, the systemic antioxidant response observed here under UV irradiation may represent a functional analog of such conserved fungal stress-signaling networks.

Together, these responses indicate that *P. locustae* adopts an energy management program characterized by enhancing repair and defense pathways while simultaneously reducing expenditures through targeted degradation of damaged proteins. Whereas previous studies of UV-induced microbial toxicity have largely emphasized DNA damage, mutagenesis, or photoreactivation repair mechanisms (25, 26), our findings highlight an additional layer of adaptation centered on metabolic and translational reprogramming. Collectively, these results suggest that obligate parasitic microsporidia, despite extensive genome reduction, retain the capacity for rapid and precise stress adaptation through strategic regulation of residual metabolic and proteostatic pathways.

This study elucidates the molecular basis underlying the environmental durability of *Paranosema locustae*, revealing stress-specific proteomic responses despite its highly reduced genome. During desiccation, spores exhibit transcriptional changes that may facilitate adaptation to dry conditions. In contrast, UV irradiation induces an UPS-centered maintenance program that preserves proteome integrity under oxidative stress.

Collectively, these findings advance our understanding of stress adaptation and dormancy regulation in early-diverging fungal lineages, and highlight how obligate parasitic fungi balance metabolic restraint with rapid reactivation under fluctuating environmental conditions.

### Limitations of this study

Several limitations should be considered. First, the proteome coverage (~187 protein groups, ~7.2% of the predicted ~2,600 coding genes) is limited, so the conclusions reflect the confidently identified proteome, and low-coverage regions were not captured. This limited coverage reflects both the biology of dormant spores—which maintain a reduced, quiescent proteome dominated by abundant structural and storage proteins—and the technical difficulty of extracting protein from the thick spore wall; untargeted DIA-MS further under-samples low-abundance regulatory proteins. Future whole-transcriptome sequencing of stressed spores would complement this proteomic survey and provide a more complete view of the stress-responsive gene repertoire. Second, because several enriched pathways contained only a single DEP, the pathway-level enrichment results should be interpreted as exploratory. Third, although qRT-PCR validated transcriptional changes for selected genes, antibody-based protein-level validation (western blot) was not performed because commercial antibodies against microsporidian proteins are unavailable; however, the reported DIA fold changes are supported by multiple unique peptides per protein (Table S4; Fig. S2). Fourth, the PPI networks were inferred from *Saccharomyces cerevisiae* STRING interactions mapped onto *P. locustae* orthologs and therefore reflect conserved interactions only. Fifth, the UV-C wavelength used here differs from natural solar UV, and field validation is needed to confirm ecological relevance.

## Data Availability

The mass spectrometry proteomics data (nine RAW files: CK1-3, GR1-3, ZW1-3) have been deposited in the ProteomeXchange Consortium via the iProX partner repository with the data set identifier PXD081039 (https://proteomecentral.proteomexchange.org/). The custom *A. locustae* protein database (NCBI-Antonospora locustae [278021]-3863-20241014.fasta, 3,863 entries) and the DIA-NN output files, including the protein quantification matrix (Table S1), are available as supplemental files.

## SUPPLEMENTAL MATERIAL

Figure S1 spectrum.01803-26-s0001.tif Post-normalization protein intensity distribution across all nine samples.

Figure S2 spectrum.01803-26-s0002.tif Individual peptide-level log2 fold changes confirm the protein-level direction of change: change for the key differentially expressed proteins.

Table S1 spectrum.01803-26-s0003.xlsx Protein quantification matrix.

Table S2 spectrum.01803-26-s0004.xlsx BLASTp validation of differentially expressed proteins (DEPs) from *P. locustae* spores.

Table S3 spectrum.01803-26-s0005.xlsx qRT‑PCR validation of differentially expressed genes in *P. locustae* spores.

Table S4 spectrum.01803-26-s0006.xlsx Peptide-level evidence for key differentially expressed proteins (DEPs).

ASM does not own the copyrights to Supplemental Material that may be linked to, or accessed through, an article. The authors have granted ASM a non-exclusive, world-wide license to publish the Supplemental Material files. Please contact the corresponding author directly for reuse.
